# The complete chloroplast genome of *Phyllostachys heteroclada* f. *solida* (Poaceae)

**DOI:** 10.1080/23802359.2021.1875904

**Published:** 2021-02-12

**Authors:** Hu Ya-ping, Zhou Jie, Yu Zhao-Yan, Li Jia-Jia, Xu Ming-Ye, Guo Qi-Rong

**Affiliations:** a Co-Innovation Center for Sustainable Forestry in Southern China, Nanjing Forestry University, Nanjing, China; b International Center of Bamboo and Rattan, Beijing, China

**Keywords:** *Phyllostachys heteroclada* f. *solida*, chloroplast genome, phylogenetic analysis, sequencing

## Abstract

*Phyllostachys heteroclada* f. *solida* is a precious wood-use bamboo resource, with almost solid stem. The complete chloroplast genome of the *Phyllostachys heteroclada* f. *solida* was the first time to assemble from Illumina pair-end sequencing data in this work. The total genome size of *Phyllostachys heteroclada* f. *solida* was 156,559 bp in length, containing a large single-copy (LSC) region of 89,200 bp, a small single-copy (SSC) region of 14,876 bp, and a pair of inverted repeat (IR) regions of 23,798 bp. The overall GC content of the genome was 36.12%, and the corresponding values of the LSC, SSC, and IR regions were 36.98, 33.15, and 44.22%, respectively. A total of 136 genes were annotated, including 88 protein-coding genes, 40 tRNA genes, and 8 rRNA genes. Phylogenetic analysis results strongly supported that *Phyllostachys heteroclada* f. *solida* was closely related to *Phyllostachys reticulate*.

The culm wall of *Phyllostachys heteroclada* f. *solida*, belonging to Phyllostachys, is particularly thick, which is nearly solid in the thinner rod. *P. heteroclada* f. *solida* is mainly cultivated in Jiangsu, Anhui, Zhejiang and Hunan Provinces of China and the United States. It has been reported that the complete chloroplast genome of Phyllostachys genus has *Phyllostachys edulis* cultivar pachyloen, *Phyllostachys edulis*, *Phyllostachys nigra* var. *henonis*, *Phyllostachys reticulata* and *Phyllostachys sulphurea* (Zhang et al. 2011; Wu and Ge 2012; Cao and Gao 2016; Huang et al. 2020). In the present study, we reported the complete cp genome sequence of *P. heteroclada* f. *solida* based on Illumina pair-end data for the first time. We also explored its phylogenetic relationship with other plant species, which would help our better understanding of the evolution of Phyllostachys cp genome.

The fresh leaves of *Phyllostachys heteroclada* f. *solida* were collected from the experimental bamboo forest (113.1124063° E, 28.2698183° N, 44.9 m above sea level) in Lukou Town, Changsha County, Hunan Province, China. The voucher specimens have been deposited in the college of forestry, Nanjing Forestry University (NJFU-2020778). Total genome DNA was extracted with the Qiagen plant genomic DNA prep kit (Sangon Biotech, Shanghai, China), which were sequence using the Illumina HiSeq 2500 platform. Approximately, 67.5 GB of raw data were generated with 150 bp paired-end read lengths. The raw data were used to assemble the complete cp genome using GetOrganelle software (Jin et al. 2018) with *Phyllostachys edulis* as the reference. Genome annotation was performed with the program Geneious R8 (Biomatters Ltd, Auckland, New Zealand) by comparing the sequences with the cp genome of *Phyllostachys edulis*, coupled with manual. The tRNA genes were further confirmed through online tRNAscan-SE web servers (Schattner et al. 2005). A gene map of the annotated *P. heteroclada* f. *solida* cp genome was drawn by OGdraw online (Lohse et al. 2013). Furthermore, the cp genome data of *P. heteroclada* f. *solida* was uploaded to GenBank (https://www.ncbi.nlm.nih.gov/genbank/), and its number was MW075109.

The cp genome of *P. heteroclada* f. *solida* was a quadripartite circular with 139,667 bp, which comprised of a large-single copy (LSC) region of 83,200 bp and a small single copy (SSC) region of 12,876 bp, separated by two inverted repeat (IR) regions of 21,798 bp, respectively. The GC content of the total genome was 36.12%, whereas the IR region had a higher GC content (38.87%) than LSC (36.98%) and SSC (33.15%). The cp genome encoded 136 genes, including 88 protein-coding genes, 40 tRNA genes, and 8 rRNA genes.

In order to study the relationship between *P. heteroclada* f. *solida* and other Phyllostachys plants, the cp genome data of six species of Phyllostachys (*Phyllostachys edulis* cultivar Pachyloen, *Phyllostachys edulis*, *Phyllostachys nigra* var. *henonis*, *Phyllostachys sulphurea*, *Phyllostachys propinqua* and *Phyllostachys reticulate*), five species of Arundinaria (*Arundinaria fargesii*, *Arundinaria humbertii*, *Arundinaria gigantean*, *Arundinaria appalachiana* and *Arundinaria tecta*) have been published in the NCBI gene library were used to align by MAFFT v7.313 (Katoh and Standley 2013) and construct phylogenetic trees (Figure 1). Phylogenetic analysis results strongly supported that *P. heteroclada* f. *solida* was closely related to *Phyllostachys reticulate*.

**Figure 1.**
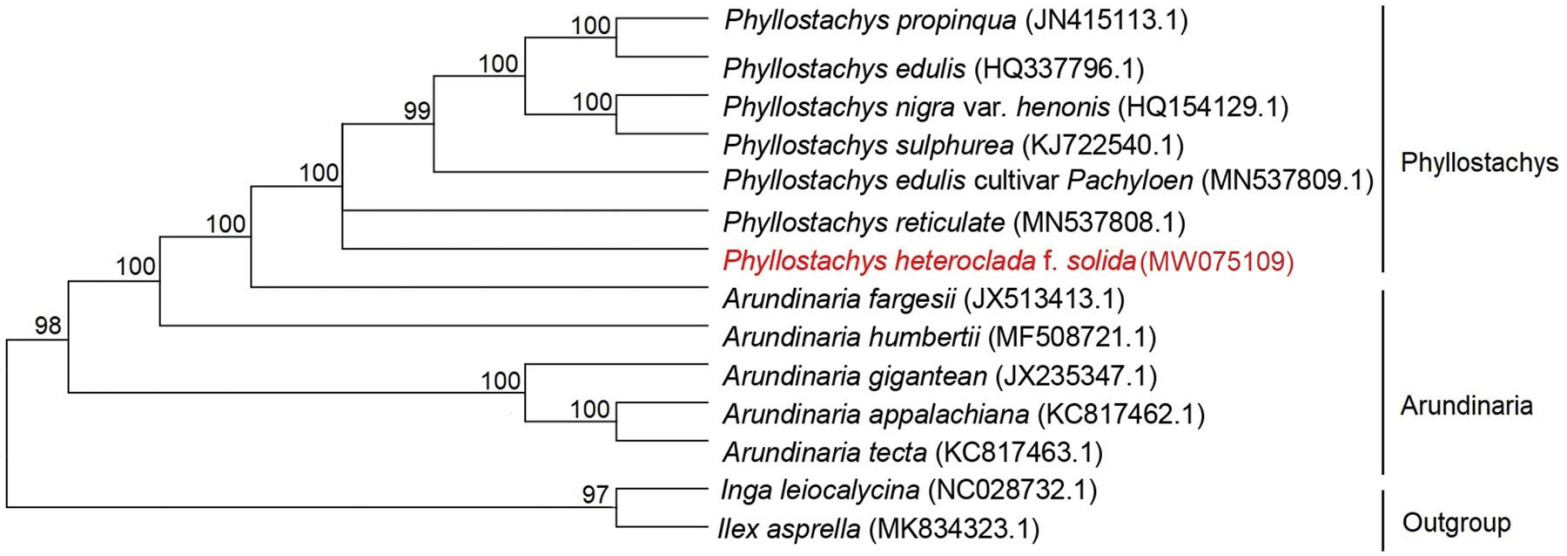
Phylogenetic relationships among 12 complete chloroplast genomes of Phyllostachys and Arundinaria. Bootstrap support values are given at the nodes.

## Data Availability

The genome sequence data that support the findings of this study are openly available in GenBank at https://www.ncbi.nlm.nih.gov/genbank/ under the accession no. MW075109. The associated BioProject, SRA, and Bio-Sample numbers are PRJNA642983, SRS6922745, and SAMN15402429 respectively in NCBI.
